# The oldest freshwater crabs: claws on dinosaur bones

**DOI:** 10.1038/s41598-019-56180-w

**Published:** 2019-12-27

**Authors:** Ninon Robin, Barry W. M. van Bakel, Matúš Hyžný, Aude Cincotta, Géraldine Garcia, Sylvain Charbonnier, Pascal Godefroit, Xavier Valentin

**Affiliations:** 1 Centre de Recherche en Paléontologie – Paris (CR2P, UMR 7207), Sorbonne Université, MNHN, CNRS, Muséum national d’Histoire naturelle, Département Origines & Évolution (CP38), 57 rue Cuvier, 75005 Paris, France; 2 School of Biological, Earth and Environmental Sciences, University College Cork, Distillery Fields, North Mall, Cork, T23 N73K Ireland; 3 Oertijdmuseum, Bosscheweg 80, 5283 WB Boxtel, The Netherlands; 4 Naturalis Biodiversity Center, P.O. Box 9517, 2300 RA Leiden, The Netherlands; 5 Department of Geology and Palaeontology, Faculty of Natural Sciences, Comenius University, Mlynská dolina G1, 842 15 Bratislava, Slovakia; 6 Laboratoire de Paléontologie, Évolution, Paléoécosystèmes et Paléoprimatologie (PALEVOPRIM, UMR7262 CNRS INEE), Université de Poitiers, 6, rue Michel-Brunet, 86073 Poitiers, Cedex France; 7 Directorate ‘Earth & History of Life’, Royal Belgian Institute of Natural Sciences, rue Vautier 29, 1000 Brussels, Belgium; 8 Palaios association, 15 rue de l’aumônerie, 86300 Valdivienne, France

**Keywords:** Palaeontology, Behavioural ecology, Freshwater ecology, Palaeoecology

## Abstract

With approximately 1,500 extant species, freshwater crabs (Decapoda: Brachyura) are among the most diverse decapod crustaceans. Nevertheless, their fossil record is extremely limited: only Potamidae, Potamonautidae and Trichodactylidae are reported up to the Eocene of the Neotropics so far. This work documents unusually large decapod claws from the Upper Cretaceous (Campanian) continental deposits of Velaux and vicinity (southern France), in close association with large vertebrate remains. In addition to (1) the systematic assignment of these claws, the study addresses (2) the salinity trends in the deposit environment from its faunal assemblage and the elementary chemical patterns of fossils, and (3) the likely scenario for their auto/allochthony in the Velaux fluvial system. These claws belong to a new taxon, *Dinocarcinus velauciensis* n. gen. n. sp., referred to as Portunoidea sensu lato, a group of “true” crabs nowadays linked to marine systems. However, the faunal assemblage, the claw taphonomy and the carbonates Y/Ho signatures support their ancient freshwater/terrestrial ecology, making them the oldest reported continental brachyurans and extending the presence of crabs in freshwater environments by 40 Ma. Either as primary or as secondary freshwater crabs, the occurrence of these portunoids in Velaux is an evidence for the independent colonizations of continental environments by multiple brachyuran clades over time, as early as the Campanian.

## Introduction

With approximately 1,500 extant species^1^, freshwater brachyuran crabs (Decapoda: Brachyura) are among the most diverse decapod crustaceans. Nevertheless, their fossil record is extremely limited. Representatives of three families were identified unequivocally as fossils, including Potamidae Ortmann, 1896^2^, Potamonautidae Bott, 1970^3^ and Trichodactylidae H. Milne Edwards, 1853^4–7^. Articulated exoskeletons of fossil freshwater crabs are rare^7–14^, although isolated cheliped fingers are much more frequent, but difficult to evaluate taxonomically^9,15–18^. Up to now, *Tanzanonautes tuerkayi* Feldmann *et al*.^13^ (Potamonautidae) from the Oligocene of Tanzania (*ca* 30 Ma) is the oldest fossil record of a freshwater brachyuran in the Old World and there is no record of Potamidae older than early Miocene^9^. The oldest record of freshwater crabs is from the middle Eocene of the Amazon Basin (*ca* 40 Ma) and belongs to the family Trichodactylidae^7^, a group of crabs that likely colonized freshwater habitats independently from potamoids, as indicated by morphology^19^ and molecular phylogeny^20^.

The present paper reports the remains of brachyuran crabs from fluvial Late Cretaceous (late Campanian; *ca* 72–74 Ma) localities of southern France (Velaux-La Bastide Neuve and vicinity), fossilized in association with vertebrate remains. Close associations of different and diverse fossil organisms may both (1) be the result of a long-distance transport of allochthonous remains or (2) testify of local biocoenoses for which members of quite restricted ecosystems have deposited altogether. These claws are of exceptional large size compared to most Late Cretaceous marine crab claws; and interestingly do not conform to the morphology of any extant freshwater crab family. The presence of presumably freshwater crabs in Campanian deposits is quite unexpected and represents the earliest record of the colonization of freshwater environments by brachyuran decapod crustaceans. It roughly doubles the previously oldest evidence of 40 Ma, and would further support the independent invasion of freshwater environment by several distinct brachyuran lineages^6,21,22^.

The study aims at (1) proposing a systematic assignment for these claws, (2) characterizing the actual salinity trend of their depositional environment, based on the channel fauna assemblage and elementary chemical patterns of fossils and (3) identifying the relevant taphonomic scenario for the presence of crab claws within a fluvial system. As all these approaches support a freshwater or terrestrial signature for the paleoenvironment of these large-clawed brachyurans; we then discuss the implications for presumed multiple invasions of freshwater habitats by crustacean decapods over time.

## Velaux-La Bastide Neuve Channel

Velaux-La Bastide Neuve is located in the western part of the Aix-en-Provence Basin, Southeastern France. K-Ar dating of the locality was attempted based on glauconites collected from sandstones^23^, but these minerals are clearly reworked from lower Aptian marine limestones and therefore useless for dating the site^23^. Magnetostratigraphic analysis of the deposits, however, correlates with the normal chron of chron 32^23^, corresponding to an age of 71.6 to 74 Ma^24^. Along with correlations with charophytes and dinosaur eggshell biozones, a late Campanian age for the locality may confidently be proposed^23,25–27^. The fossil site is mostly known for its vertebrates assemblage, recovered from three different sedimentological sequences and corresponding to newly described dinosaur (titanosaurid sauropod^26,28^; rhabdodontid ornithopod^29^) and pterosaur (azhdarchid pterosaur^30^) taxa, as well as eusuchian crocodilians^27^. Apart from the diapsids, vertebrates consist of disarticulated pleurodiran and cryptodiran turtles, disconnected remains of sarco-/actinopterygians, and chondrichthyan teeth. Freshwater bivalves (*Unio*) and gastropods (*Physa*, *Melania*)^30^, macro-remains of angiosperm plants and charophytes complete the whole fossiliferous assemblage together with the herein described crustacean remains. The lithological section consists of 16.3 meters of alternating sandstones, siltstones – including palaeosols – and mudstones. Lacustrine limestones occur in the uppermost part of the section. The succession was deposited in a fluvio-lacustrine environmental setting. The sedimentology of the site together with the fossil assemblage indicates a likely freshwater setting for the deposits. The succession of conglomeratic sandstones, siltstones (including palaeosols), mudstones and lacustrine limestone on top of the stratigraphic section indicate sedimentation in, respectively, a low-energy fluvial channel, channel levees, alluvial plain and lake^23^. Given the proximity of Velaux to the palaeo-coast during the Late Cretaceous^31,32^, occasional marine incursions are not excluded, even though they were not recorded at Velaux-La Bastide Neuve nor at other fossiliferous localities of the same age in the region^23,25^.

## Results

The studied material (Tab. 1) consists of seven (partial) claws and associated vertebrate remains collected from sequence 2 of the sedimentary succession of Velaux-La Bastide Neuve locality and one from the close locality of Rognac-Les Frégates (about four km from Velaux, corresponding to similar layers). Specimens are housed in the palaeontological collections of Velaux municipal institutions (Musée du Moulin Seigneurial/Velaux-La Bastide Neuve: MMS/VBN.00.004, 09.69e, 12.A.006, 02.94, 09.43, 09.132d, 12.A.003) and of the Muséum d'Histoire naturelle d'Aix-en-Provence, France (MHN AIX PI 1991.1, coll. Valentin).

### Systematic palaeontology

#### The crabs

**Preliminary remarks**: Taxonomic assignment of isolated claws of brachyuran crabs at the species or genus level is difficult, if not impossible in many cases. If direct comparisons with extant taxa is straightforward and often helpful for identifying Pliocene and Pleistocene brachyurans^33–36^, this task becomes more complicated when working with material of Miocene age^37–40^. The taxonomic evaluation of isolated fossil cheliped fingers is of course further challenging. Erecting new taxa based on isolated brachyuran chelae alone has not been attempted yet, although this method is regarded as valid in other decapod groups, including paguroid hermit crabs^41–43^, erymid lobsters^44,45^ or callianassid ghost shrimps^46,47^. If working with distinct claw morphologies, the erection of new taxa can be done^48^. Alternatively, parataxonomy can be used^49^.

We assume that the morphology and also the size of the studied claws from the two localities Velaux-La Bastide Neuve and Rognac-Les Frégates are distinct enough to warrant the validity of the new form genus *Dinocarcinus*. Based on the general morphology of its claws, we include this form genus within Portunoidea sensu lato^50^. For portunoid crabs the presence of large proximal molariform tooth on the dactylus on one of the chelae, and serial (often bi- and tri-lobed) conical teeth on the dactylus and fixed finger of both chelae are typical^50,51^. It should, however, be mentioned that the presence of a massive molariform tooth is not unique for portunoids, but it can be found in a number of heterotreme superfamilies (sensu Ng *et al*.^52^). Our material shows some affinities to selected representatives of Xanthidae, although, in these crabs, finger usually is somewhat shorter than manus^53^ and generally exhibit a different pattern of serial teeth^50^. In this respect, the *Dinocarcinus* material is not preserved sufficiently enough to allow detailed comparison of the cheliped dentition of various portunoid or xanthoid crabs. Yet, its taxonomic features (as mentioned above) point out affinities with portunoids, although this attribution has to be considered as preliminary. In this sense, *Dinocarcinus velauciensis* is kept in open nomenclature.

**Figure 1:**
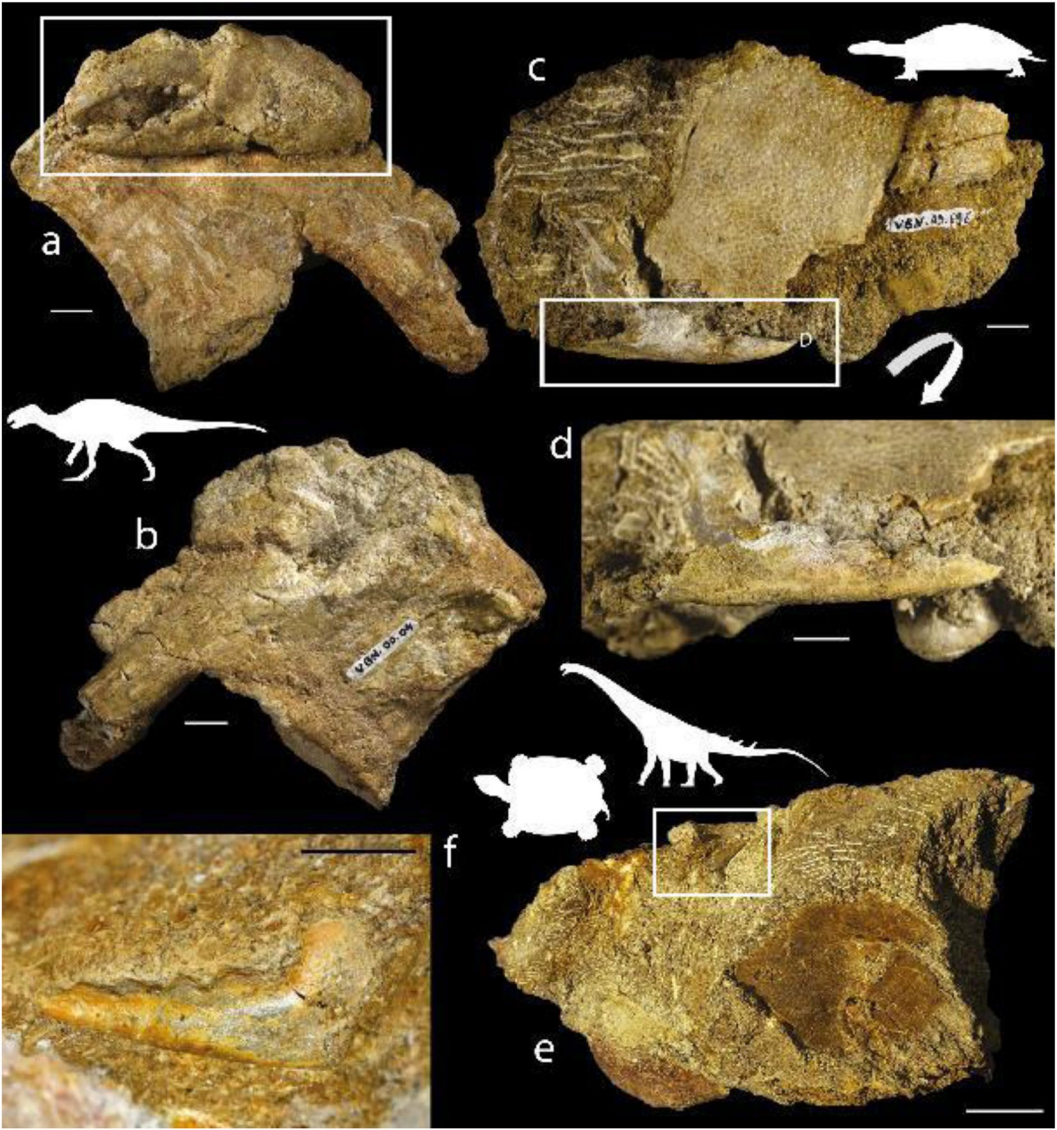
*Dinocarcinus velauciensis* Van Bakel, Hyžný, Valentin & Robin n. gen., n. sp. Claws associated with vertebrate remains. (**a,b**) MMS/VBN.00.004, holotype: anterior (**a**) and posterior (**b**) views of the claw-bearing ornithopod (rhabdodontid) vertebra, (**c,d**) MMS/VBN.09.69e: outer view (**c**) and close-up (**d**) of the close sedimentary association of the claw with a turtle (solemydid) plastral plate, (**e,f**) MMS/VBN.12.A.006: outer view (**c**) and close-up (**d**) of the block association of the claw with a turtle (bothremydid) plastral plate, ornithopod (rhabdodontid tooth) and partial sauropod (titanosaurid) dorsal vertebra with its ossified tendon. Scale bars = 1 cm (**a–d,f**); = 3 cm (**e**). Photographs. L. Cazes.

**Figure 2:**
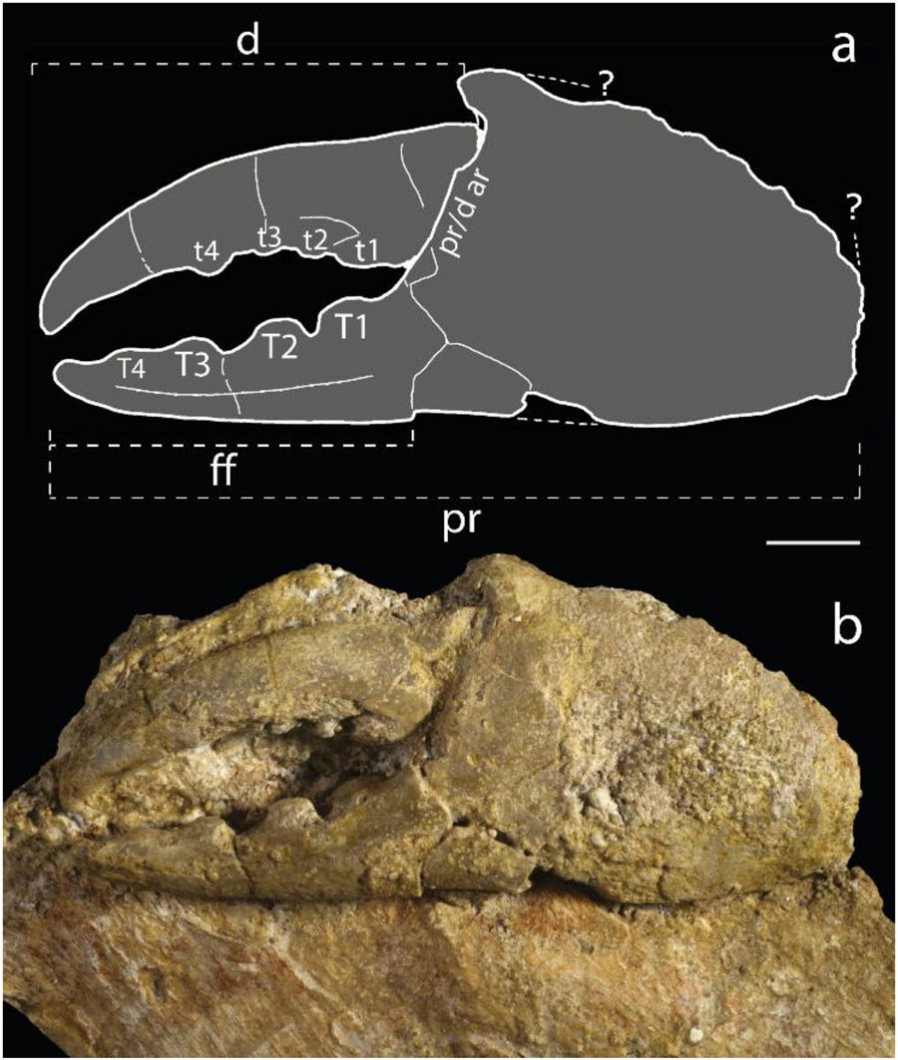
*Dinocarcinus velauciensis* Van Bakel, Hyžný, Valentin & Robin n. gen., n. sp. (**a**,**b**) Illustrative features of holotype MMS/VBN.00.004 (complete chela). (**d**) = dactylus; ff = fixed finger; pr = propodus; pr/d ar = propodus/dactylus articulation; t1-4 = dactylus teeth; T1-4 = fixed finger teeth;? = questionable limits. Scale bars = 1 cm. Drawing. B. van Bakel, photograph. L. Cazes.

**Figure 3:**
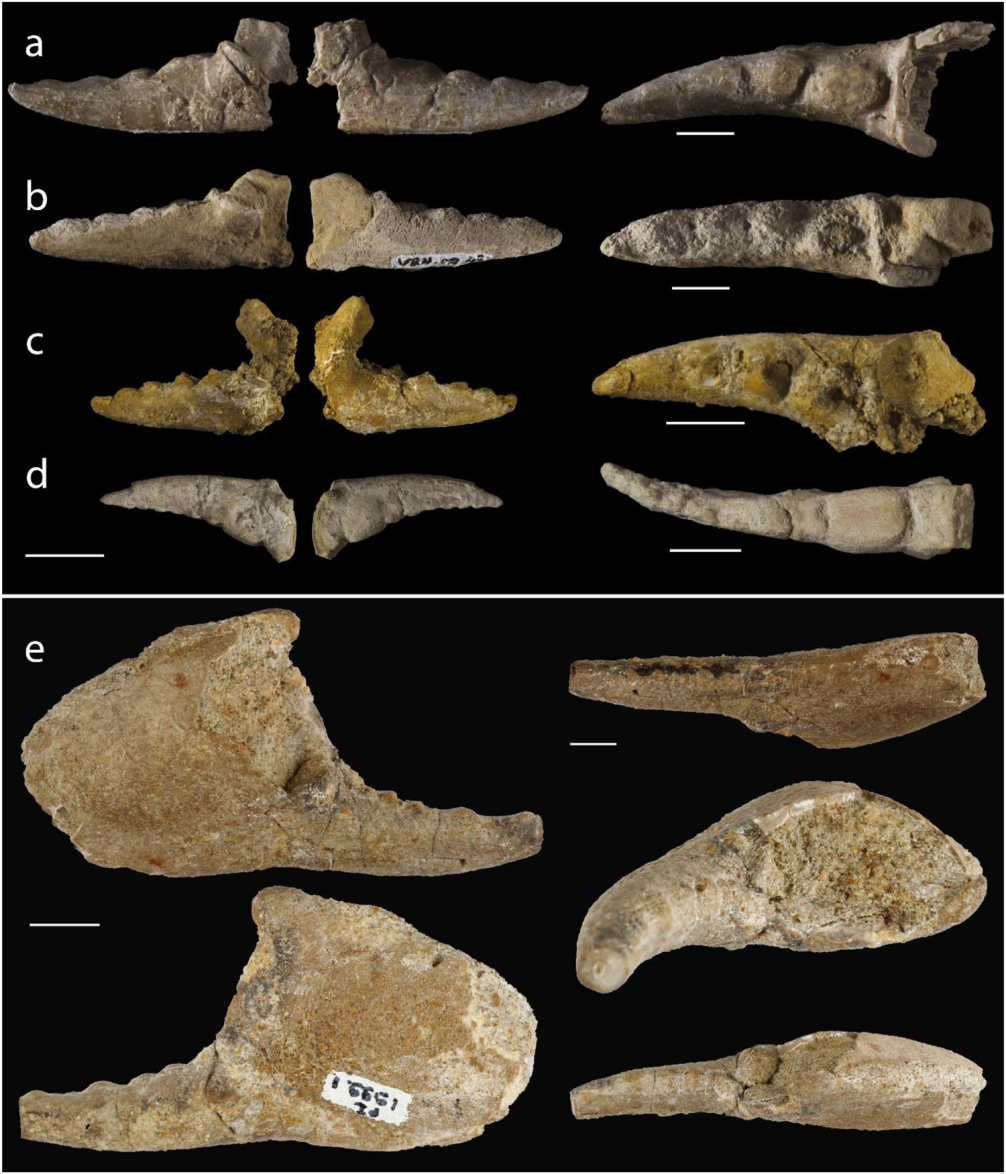
*Dinocarcinus velauciensis* Van Bakel, Hyžný, Valentin & Robin n. gen., n. sp. Isolated claws found scattered in sediment. (**a–d**) From left to right: inner, outer and occlusal views of dactyli showing teeth arrangement. (**a**) MMS/VBN.02.94, (**b**) MMS/VBN.09.43, (**c**) MMS/VBN.09.132d (**d**) MMS/VBN.12.A.003. (**e**) MHN AIX PI 1999.1 (coll. X. Valentin) from the Campanian of Rognac, 4 km from Velaux. From up to down and left to right: inner, outer, marginal and occlusal two views showing teeth arrangement. Scale bars = 1 cm. Photographs. L. Cazes (**a–d**), Y. Dutour and E. Turini (**e**).

Portunoidea sensu lato (see above) *Dinocarcinus* n. gen. Van Bakel, Hyžný, Valentin & Robin Figs. 1, 2 and 3.

**Etymology**: Denoting the actual association with dinosaur (ornithopodan) remains.

**Type species**: *Dinocarcinus velauciensis* n. gen. n. sp.

**Diagnosis**: Chelae large and massive. Fingers gaping, arched, with strong teeth, proximal tooth molariform. Fixed finger dorsal surface with single “pitted groove”, palm surface smooth, articulation with dactylus oblique, prominent.

**Remarks**: The morphology of the claws, namely heavily calcified fingers, strong molariform teeth and grooved fingers, are typical for several eubrachyuran crabs. There are only few representatives of podotreme clades which grew to large sizes, with particular exceptions of Dakoticancridae and Cenomanocarcinidae, which could have claws comparable in size to *Dinocarcinus velauciensis* n. gen. n. sp. Figured claws of *Avitelmessus* Rathbun, 1923^54^, show the following^54–56^: the fingers’ (distal) teeth are not molariform, fingers are less robust and less strongly calcified than the remains studied herein. The claws of *Avitelmessus* are more curved, the palm is longer than the fingers, both palm and fingers have crests and grooves; the fingers tips are hooked; distinguishing it easily from *Dinocarcinus* n. gen. The palaeocorystoid Cenomanocarcinidae could attain large sizes and had massive claws. The claws of *Cenomanocarcinus* Van Straelen, 1936^57^ are figured by Guinot *et al*.^58^ (Fig. 6) and are characterized by spinose claws, flattened in cross section, a slightly downturned fixed finger, spines along the upper margin of the claw and dactylus, and hooked tips. Compare also the very large claws of ‘*Oncopareia’ heterodon* Bosquet, 1854^59^, now considered to be a palaeocorystoid (in Jagt *et al*.^60^: plate 5). As discussed above, the claw morphology of *Dinocarcinus velauciensis* n. gen. n. sp. does not match that of the Dakoticancroidea, Palaeocorystoidea, or any known Podotremata.

Within Eubrachyura, the robust, strongly calcified fingers, overall claw shape, and molariform teeth, match that of the Portunoidea. This large group of overall large-sized crabs have several Mesozoic occurrences, and some of them in large sizes. *Ophthalmoplax* Rathbun, 1935^61^, now considered a representative of Macropipidae^62^ has a great size range, from very large *Ophthalmoplax brasiliana* Maury, 1930^63^, to rather small *O. minimus* Osso^64^. Their claws [compare^65^ (Figs. 3.2, 3.3, 4.2, 4.13, 4.14) with^66^ (Fig. 6.7)] are spinose, keeled, with major claws showing a large bulbous proximal tooth index inferred to be used for shell breaking^67^. These specialized claws can be easily distinguished from the more simple, unarmed claws of *Dinocarcinus* n. gen. *Eogeryon* Osso^66^ (Cenomanian of Spain) is assigned to the Portunoidea in its own family (Eogeryonidae Osso^66^). Geryonidae Colosi, 1923^68^ and Eogeryonidae are considered as early diverging families within Portunoidea. *Eogeryon* is characterized by large claw size relative to the carapace, equal ratio palm-fingers, with strongly calcified fingers with molariform teeth, and grooved fixed finger. Its claw morphology is typical of that of Portunoidea, and compared with that of *Styracocarcinus meridionalis* (Secrétan, 1961^69^) from the?Campanian of Morocco. Claws of *Litoricola macrodactylus* (Van Straelen, 1924^70^) from the Paleocene of southern France and Northern Spain, are highly comparable with those of *Dinocarcinus* n. gen., however they show a bulbous proximal crushing tooth on the dactylus of the major claw. Also, the fingers in *Dinocarcinus* n. gen. are more gaping than those of *Litoricola*.

The claw morphology of *Dinocarcinus* n. gen. shows few diagnostic characters for superfamily level assignment (Portunoidea), namely heavily calcified claws, a grooved fixed finger, molariform teeth, palm, and fingers subequal in length, and blunt, non-hooked fingertips (Fig. 2). More accurate assignation is not possible at this point. An early diverging position within Portunoidea is possible considering morphology, geologic age, large size, and similar families occurring at that time.

Of note is yet another occurrence of the Late Cretaceous crab, namely *Megaxantho zoque* Vega *et al*., 2001 from the Maastrichtian of Mexico^71^. Its sheer size and general morphology of chelae is reminiscent of *Dinocarcinus*; both taxa, however, differ from each other in a number of characters. Cheliped dentition of *Megaxantho* was directly compared to portunoids^67,71^. Originally, *Megaxantho* was classified within Xanthidae; nevertheless, its attribution was later questioned and its assignment to Xanthoidea incertae sedis was finally suggested^5^.

*Dinocarcinus velauciensis* Van Bakel, Hyžný, Valentin & Robin n. sp. Figures 1, 2 and 3.

**Type material**: Holotype: MMS/VBN.00.004; Paratypes 1–4: MMS/VBN.02.94, 09.132d, 12.A.003, 12.A.006 (see Table 1).

**Table 1:** Examined fossil material and preservation state.

Catalogue	Number	Item	Preservation	Type status
MMS/VBN.	00.004	left-hand claw, with dactylus	well preserved	Holotype
MMS/VBN.	09.69e	right-hand fixed finger	moderately preserved	/
MMS/VBN.	12.A.006	right-hand fixed finger	moderately preserved	Paratype 4
MMS/VBN.	02.94	right-hand fixed finger	well preserved. very large	Paratype 1
MMS/VBN.	09.43	right-hand fixed finger	coarse, surface partly dissolved	/
MMS/VBN.	09.132d	right-hand fixed finger	well preserved	Paratype 2
MMS/VBN.	12.A.003	right-hand dactylus	moderately preserved	Paratype 3
MHN AIX	PI 1999.1	left-hand claw	well- preserved	/

**Etymology**: From Velaux-La Bastide Neuve, Bouches-du-Rhône, the type locality.

**Diagnosis**: As for genus.

**Description**: Only claws known; claw very large (approximately 85 mm for holotype MMS.VBN.00.004), massive, outer surface flat. Palm subrectangular, slightly longer than high, slightly longer than fixed finger. Fixed and movable fingers inwards curved, clearly gaping. Lower propodus margin curved, weakly convex. Upper (cutting) margin of fixed finger straight. In total, 4 strong teeth on fixed finger. Proximal tooth massive (T1/t1), molariform in both fingers. Surface of molariform proximal tooth bulbous on fixed finger, flat on dactylus. Fixed finger dorsal surface with a single “pitted groove”. Articulation dactylus-propodus prominent, oblique. Both finger tips sharp, pointed. Dactylus upper and lower margin curved in dorsal view; dactylus cross-section flatter than propodus cross-section. Dactylus more strongly curved in dorsal view than fixed finger. Dactylus margin at approximately 100 degrees at upper (cutting) margin of fixed finger. Fingers strongly calcified, cuticle surface (as preserved) covered with microscopic dense, flattened granules.

#### The associated vertebrates

Four of the brachyuran claws were discovered in close association with vertebrate remains. The most complete and larger specimen (MMS/VBN.00.004) is fossilized onto a vertebra that closely resembles a posterior cervical vertebra of a rhabdodontids Iguanodontia (Fig. 1a,b). Rhabdodontids are represented at Velaux by the genus *Matheronodon* Godefroit *et al*.^29^ and in other Late Cretaceous localities from southern France, by *Rhabdodon* Matheron^72^. MMS/VBN.09.69e is found in close sedimentary association with the plastral plate of a terrestrial turtle (*Solemys* de Lapparent de Broin & Murelaga^73^, Solemydidae) (Fig. 1c,d). MMS/VBN.12.A.006 is preserved in a 50 cm large block that also contains a turtle plastral plate (*Polysternon* Portis^74^, Bothremydidae), a rhabdodontid tooth and centrum, as well as hybodontid shark teeth (Fig. 1e,f). MMS/VBN.12.A.003, figured isolated (Fig. 3d), has been extracted from a comparable block, also containing a crocodylomorph skull, a rhabdodontid tooth, as well as a partial titanosaurid dorsal vertebra showing preserved ossified tendons.

### Freshwater environment

#### Taxonomic/ecological diversity in the sequence 2 of the channel

**Figure 4:**
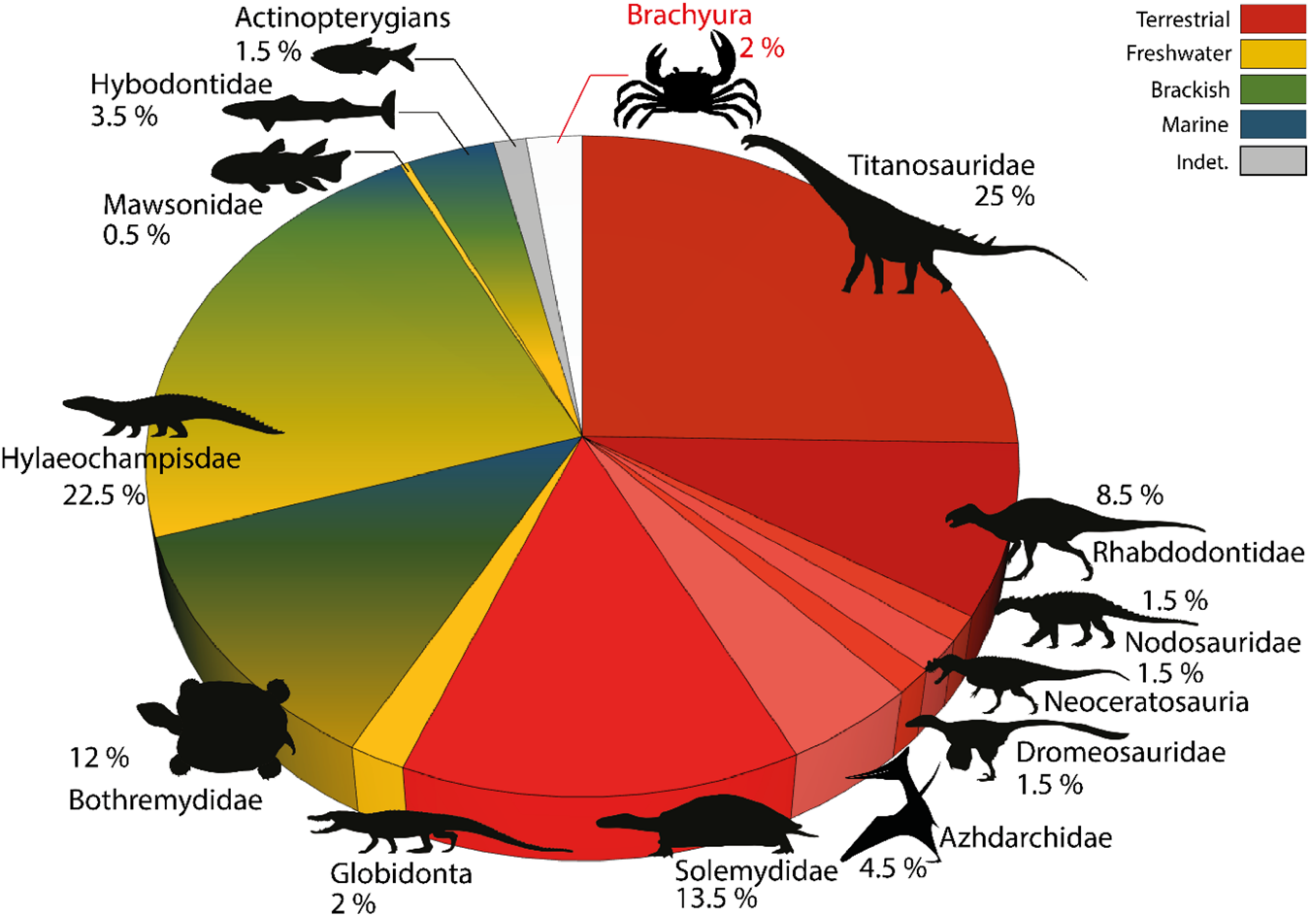
Distribution of the taxa remains (n = 308), recovered from the sequence 2 and associated described depositional environments (see *III. 2a. Taxonomic/ecological diversity in the sequence 2 of the channel*), based on Velaux-La Bastide Neuve excavation campaigns. Diagram N. Robin, G. Garcia, X. Valentin.

The freshwater palaeoenvironment of the channel in sequence 2 is strongly supported not only by sedimentological evidence^23^, but also by the most recently collected taxonomic assemblage itself. The fossil remains (n = 308 specimens) consist of 42.5% of strict terrestrial/aerian Avemetatarsalia indicative of an absolute continental faunal assemblage (Fig. 4). Aquatic and semi-aquatic taxa consist of families and genera, whose previous depositional record is strongly anchored in freshwater environments. Among archosaurs, the hylaeochampisdae crocodylomorph *Allodaposuchus* Nopsca^27,75^) has so far been reported from fluvial inner/lacustrine-interpreted environments^76–78^ and once in a more coastal swampy area^79^. These are associated to a small amount of Globidonta, which, as members of Alligatoroidea, would have secondarily lost salt glands and therefore have been also restricted to freshwater settings^80^. Other highly abundant sauropsids in Velaux are chelonians, equivalently represented by Bothremydidae (*Polysternon*) and Solemydidae (*Solemys*, Fig. 4). If the former family is recognised as the most abundant and diverse European group of freshwater and coastal turtles in the uppermost Cretaceous^81^, *Polysternon* is only reported from estuarian to alluvial sediments and its sister-genus *Foxemys* Tong *et al*.^82^, is exclusively known from freshwater localities^83,84^. The case of Solemydidae is even more compelling because their dermal skeleton (skull osteoderms) is highly supportive of a strict terrestrial life habit^85^ rather than any degree of amphibious lifestyle. The identified chondrichtiyan teeth correspond to a unique hybodontid genus: *Meristonoides* Case & Capetta^86^, which presence at Velaux has been briefly questioned (Cuny pers. comm. in^87^), although it is well accepted that hybodontid sharks are common in fluvial ecosystems in the Cretaceous^88^. The least abundant remains at Velaux belong to an aquatic sarcopterygian identified as *Axelrodichthys megadromos* Cavin *et al*.^89^ (Cavin pers. comm.). The only known occurrence of this mawsoniid coelacanth is from another French Campanian lacustrine deposit^88^, confirming the unequivocal freshwater nature of the fauna from the sequence 2, which includes the brachyuran claws in the Velaux-La Bastide Neuve channel (Fig. 4).

#### Y/Ho ratios

As for rare-earth-elements, the Y signature of limestones and carbonate concretions can be used as a recorder of ancient seawater signatures^90–92^. Y and Ho concentrations can be compared because these elements are chemically similar in charge and ionic radius, and suggested to evolve similarly over time in few terrigenous diagenetic environments^91^. Consequently, measured on fossil carbonates, Y and Ho concentrations can partly document their relative original intake into abiogenic/biogenic carbonates and therefore inform on marine/non marine pre-diagenetic environments of formation. The carbonates of MMS/VBN.09.69e-claw display a Y/Ho ratio of 33.06 (Y = 8.61 ppm; Ho = 0.26 ppm). The ratio in MMS/VBN.09.69e-sediment is a bit lower with 29.86 (Y = 11.3 ppm; Ho = 0.37 ppm). Marine waters are known to have a quite constant Y/Ho ratio around molar 90–110, decreasing with depth^92,93^. Consequently, neither the sandstones hosting the claws, nor the claw carbonate could have been deposited and/or formed in marine conditions. Apart from the marine realm, Y/Ho data characterizing formally typical estuarine or fluviatile environments are hitherto not commonly reported^94^. Nozaki *et al*.^94^ evidenced from the study of Japanese fluvial systems that Y and Ho concentrations were constantly decreasing with the salinity, with Ho removed from seawater twice as fast as Y owing to differences in surface complexation behaviour. Unfortunately, Y and Ho absolute concentrations (which we could expect to interpret from the studied material) depend on biological and taphonomic factors of integration that simply cannot be estimated here. Consequently, the salinity of the studied channel cannot be assessed from the chemistry. In all cases, the observed Y/Ho ratio formally excludes a marine/costal pattern and seems to distinguish strongly from it.

### Taphonomy of the assemblage

**Figure 5:**
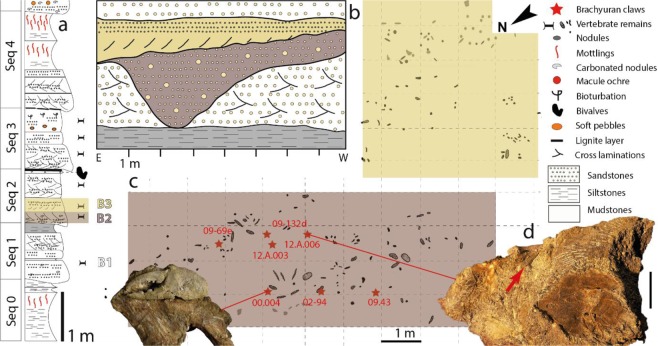
Distribution of the fossil assemblage in the deposit of Velaux-La Bastide Neuve, France. (**a**) Lower portion of the Velaux log (Seq 0–4). (**b**) Schematic spatial extension of the Velaux channel showing the distribution of bonebeds 2 (B2) and 3 (B3) in section. (**c**) Top view of the distribution of claws/bones on the excavation site, the cropping lower part of the sequence 2. (**d**) Bloc assemblage of claws (small arrow) among vertebrate remains, scale bar = 10 cm (**d**). Modified from Cincotta *et al*. 2015 (**a**), field surveys from G. Garcia & X. Valentin (**b,c**), redrawn by N. Robin, photographs. L. Cazes and X. Valentin (**d**).

The crab chelae studied herein were recovered from the fluvial channel sediments from sequence 2, which correspond to lenticular conglomeratic sandstone (Fig. 5a,b). This association of elements belonging to diverse – aquatic and terrestrial – vertebrate taxa probably results from the transport of decayed carcasses originating from diverse environmental settings in a river channel. The associated bones and tooth elements are found disarticulated. The preservation of some complete bony elements, like crocodylomorph skulls, argues for their relatively short timing of decay and transport, consistent with the most common reports of the group in fluvial/ inner lacustrine type of environments. The Velaux taphonomy would indicate a local riverine system with a low-enough energy to allow the deposit of small millimetric elements like *Meristonoides* teeth^23^. An option could be that large elements (large appendicular bones, carapace portion and skulls) would have acted as obstacles for smaller ones in a more intermediate-energy flow configuration, resulting in a mixture of elements of different sizes and spatial origins^23^. In both cases, as in any continental water system, the deposit must have occurred from upstream to downstream implying that all the remains in sequence 2 must have belonged either to original local fluvial living-individuals (sedimentary context) or to upstream/even more terrestrial ones (floodplain and levees). Consequently, the presence of strictly freshwater lineages (Globidonta crocodylomorphs), would restrict the salinity inside this part of the channel to a minimum, implying that crabs must have been living either in terrestrial or freshwater aquatic habitats. The spatial distribution of the crabs within the conglomeratic sequence (sequence 2 on Fig. 5c,d) is heterogeneous: they are in most cases horizontally spaced by several dozens of centimeters. The absence of further connection of the brachyuran remains (e.g. with manus/carpus) or other body parts than dactyli and/or propodi is poorly informative on transport/exposure time experienced by claws given the admitted proclivity of chelae from decapod crustaceans to preserve the best after years (see^95,96^ for brachyurans). Additionally, the sand-/siltstones that yielded the claws do not provide high probabilities for carapaces nor full exoskeletons to be preserved in connection (as mudstones might have for instance).

## Discussion

### Decapod crustaceans in Cretaceous continental assemblages

Decapod crustaceans, such as shrimps and ghost shrimps, have been mentioned in assemblages of continental vertebrates, including dinosaurs^97–99^. The oldest report involving brachyuran crabs is from a Cenomanian mangrove coastal environment (Bahariya Formation, Egypt)^100^. In this case, the authors even suggested a scavenging behaviour for these crabs based on their relative proximity to bony remains. Likewise, the Velaux-La Bastide Neuve channel may have provided a favorable environment for the rise of invertebrate scavengers. However, neither the claws shape nor their important size could inform on a specific diet, nor on crabs interactions with reported members of this ecosystem, although some modern freshwater durophagous crabs have dentition developed in a similar fashion^101,102^. Interestingly, it is worth stressing that consumption behaviours of decapod crustaceans by megaherbivorous dinosaurs have been reported from the Campanian of two North American formations^103^. In that case, *in-situ* coprolites of ankylosaurs, brachylophosaurs and neornithischians revealed undetermined crustacean cuticles associated to fragments of likely rotted wood fragments. This led the authors to suggest these crustaceans were consumed when they were sheltering inside dead plant logs. The cuticles could not be identified as belonging to a specific order, but were mentioned as possibly corresponding to claws reported from surrounding continental middle Campanian formations^104–106^. However, the figured corresponding claws, referred as large freshwater crab ones, clearly actually correspond to anomuran – and no brachyuran – chelae^105^ (see p. 583, Fig. 26.28). However, smaller (up to 3 cm) brachyuran-like dactyls are mentioned in the Maastrichtian of Fox Amphoux (Var, France)^106^ and the Hateg Island (Transylvania, Romania)^107^. In the latter case, the authors question the allochthonous marine origin of the claws, moved in as preys, by other animals. The consistent autochthony of all other invertebrates in the channel (*Unio*, *Physa, Melania*), and the Y/Ho carbonates displayed by claws, limit our interpretation to the most parsimonious assertion: it is rather unlikely that claws were moved into the Velaux fluvial system, as preys, from further marine environments.

### Decapod crustaceans in freshwater habitats

Decapod crustaceans inhabit virtually all water-influenced habitats, including freshwater bodies, from streams and rivers to ponds and lakes, and even caves. In fact, representatives of a number of originally exclusively marine decapod clades have successfully invaded freshwater and/or terrestrial habitats. In this respect, the following listing of freshwater decapods is not meant to be exhaustive but rather illustrative of the independent colonization of freshwater habitats within this group of crustaceans.

Among caridean shrimps, more than 650 species, making a full quarter of all described species, inhabit freshwater^108^, with representatives of *Merguia* Kemp^109^, being semi-terrestrial^110^. With approximately 650 species, virtually all crayfish are freshwater animals^111^. Many axiideans and gebiideans are able to tolerate pretty low salinity conditions. The callianassid *Lepidophthalmus* Holmes^112^, is even able to tolerate freshwater environments^113^ and *Lepidophthalmus turneranus* (White, 1861^114^) has been reported to migrate up rivers in West Africa^115^. Among anomurans, a rather speciose family Aeglidae Dana, 1852^116^, is strictly freshwater^117–119^. The majority of freshwater decapod crustaceans, however, consists of Brachyura with one fifth (>1 280 species) of all^120^.

Primary (so called *true*) freshwater crabs are those that have adopted freshwater, semi-terrestrial or terrestrial modes of life, and are able to complete their life cycle independently of the marine environment^1^. However, there are a number of brachyuran crabs able to live in freshwater habitats that include euryhaline species or secondary freshwater species from primarily marine brachyuran families^1^. These do not have direct development in their life cycle, which is typical for true freshwater crabs. Today there are five primarily freshwater families of brachyuran crabs with no marine species^120^, i.e. Gecarcinucidae, Potamidae, Potamonautidae, Pseudothelphusidae, and Trichodactylidae, whereas there are also numerous secondary freshwater, semi-terrestrial and terrestrial species among Majoidea (Hymenosomatidae), Goneplacoidea (Goneplacidae), Grapsoidea (Gecarcinidae, Sesarmidae, Varunidae) and Ocypodoidea (Ocypodidae)^1^. Many grapsoids invade or even wholly inhabit freshwater habitats. Some varunids, including representatives of *Eriocheir* De Haan, 1835^121^, and *Varuna* H. Milne Edwards, 1830^122^, not only enter estuaries, but are also found further up in rivers^1^. Sesarmids (*Sesarmoides*, *Labuanium*, *Karstarma*) can be completely adapted to freshwater, the latter being semi-terrestrial^120,123–125^, whereas *Geosesarma* De Man, 1892^126^ is found in terrestrial habitats^118,119^.

All primary freshwater crabs are entirely free from the need to enter seawater. As for the fossils from Velaux and surroundings newly described herein, it cannot be decided unequivocally whether representatives of *Dinocarcinus* n. gen. were able to complete their life cycle in the freshwater habitat or/and had direct development. The sheer size and number of claw fragments, however, may prove that crabs from the Late Cretaceous of Velaux were not only an occasional element of the respective environment, but rather a natural part of the assemblage suggesting that they were fully adapted to freshwater environment. Apparently, the remains belonged to adult animals, so it is more likely they were primary freshwater crabs.

### Freshwater decapods in the fossil record

Fossil freshwater decapods are exceedingly rare in comparison to their marine relatives. Fossil crayfish are represented only by a handful of occurrences reported so far (^123,124,127–131^ and references therein), the oldest possibly coming from the Triassic of Utah128. Fossils of freshwater caridean shrimps are similarly rare^127,129,132–135^, the oldest being reported from the Early Cretaceous of Spain^129^ and China^135^. Interestingly, the only fossil representative of nowadays strictly freshwater anomurans, the family Aeglidae, comes from marine strata^136^. Fossil freshwater brachyurans are limited to a number of occurrences of isolated claws^14,20–23^ and several fossils exhibiting preserved carapace^12–18,137^. The oldest occurrences of undisputed primary freshwater crabs is from the middle Eocene (late Lutetian/early Bartonian) of the Amazon Basin, recently reported by Klaus *et al*.^7^ who described isolated claw elements of Trichodactylidae. A recent report of *Alontecarcinus buratoi* De Angeli & Caporiondo, 2019^137^ from the middle Eocene (Bartonian) of Italy, being interpreted as the oldest representative of Potamidae, should be taken with caution because of its rather unique carapace sculpture, unknown in potamids (Sebastian Klaus, pers. comm 2019). Moreover, *A. buratoi* is described from a setting suggesting a brackish environment, which contradicts the ecology of at least extant primary freshwater crabs (as discussed above). In this respect, *Dinocarcinus velauciensis* n. gen. n. sp. reported herein is the oldest occurrence of freshwater brachyuran crabs, exceeding previous reports by approximately 40 million years. For now, it is unclear whether *Dinocarcinus* belonged to primary or secondary freshwater crabs. It is, however, of note that recent advances in resolving the phylogeny of primary freshwater crabs suggest their early divergence in brachyuran evolution^138^. Both, potamoid and portunoid crabs, i.e. primary freshwater and marine crabs, are suggested to be roughly equally old.

### Multiple invasions into freshwater habitats

From the discussion above it is clear that several lineages of decapod crustaceans independently invaded freshwater habitats, including dendrobranchiatans^139^, carideans, axiideans, astacideans, anomurans and brachyurans^11,24,118,140^. The enigmatic *Tealliocaris* Peach, 1908^141^, considered by some authors as a decapod crustaceans^142^ (but see also^143^), might represent yet another freshwater lineage. And among carideans, at least palaeomonoid, atyoid, and alpheoid shrimps independently invaded freshwater environments^108^. Moreover, presumed multiple invasions of freshwater habitats by some *Macrobrachium* Bate, 1868^144^ shrimps were also suggested^145^. The sparse fossil record of freshwater shrimps does not allow relevant time estimation of colonization of freshwater habitats; however, fully freshwater shrimps are known from the Early Cretaceous (Barremian) onward^129,135^. Crayfish represent a monophyletic group^146^ with the oldest fossil representatives known from the Late Triassic of Utah^128^. As for brachyuran freshwater crabs, there are two independent lineages. The Old World primary freshwater crabs are monophyletic^11,24,120,147^, whereas Neotropical Trichodactylidae have a separate phylogenetic origin^24,148^. Based on the morphology of its chelae, *Dinocarcinus velauciensis* n. gen. n. sp. cannot be referred to any of the extant primary or secondary freshwater families mentioned above suggesting that it may represent yet another independent “attempt” to colonize freshwater environment besides the two primary freshwater crab clades recognized today, i.e., Potamoidea and Trichodactyloidea^24,120^. Interestingly, the oldest fossil representatives of both clades come from the middle Eocene^12,137^, although the oldest putative potamid^132^ needs further investigation (see above). The geographic distribution of modern primary freshwater crabs speaks for independent invasions of the limnic habitat rather than for a Gondwanan vicariance^11,149^, contrasting with the diversification of crayfishes: the fossil record and modern distribution of the latter clade can be explained by the breakup of Pangaea and disassembly of Gondwana and Laurasia^140^. The present occurrence of *Dinocarcinus* further supports independent colonization of freshwater habitats by brachyurans. Based on molecular clock estimates, Daniels *et al*.^149^ suggested that the radiation of Afrotropical freshwater crab taxa occurred during the Early Cretaceous, whereas the age of the African Potamonautidae clade was given with 75–73 Ma (Campanian). From the fossil record of the modern freshwater families alone, such timing cannot be apprehended; however, the discovery of fossil crabs from Velaux-La Bastide Neuve illustrates that brachyuran crabs attempted to colonize freshwater habitats in the Old World at least from the Campanian onwards.

One of the key processes driving freshwater crab diversification is likely allopatric speciation resulting from geographic isolation, often coupled with habitat heterogeneity and numerous ecological niches and microhabitats resulting from the complicated topography and hydrology of freshwater environments^1^. During the Campanian, *Dinocarcinus velauciensis* inhabited Europe, which was at its time an archipelago rather than a proper landmass^37,64,75,136,150^. Based on the material from Velaux and Rognac described herein, we suggest that the freshwater habitats of islands in the Tethyan epicontinental sea were colonized by marine portunoids during the Late Cretaceous. Nowadays, most secondary freshwater brachyurans have a marine larval development and would reach inland habitats more likely as adults. This might also have been the case for *Dinocarcinus velauciensis*.

## Conclusions

*Dinocarcinus velauciensis* n. gen. n. sp. from the late Campanian of Southern France, belongs to Portunoidea sensu lato, a group of “true crab” that are nowadays intimately linked to marine systems. The sedimentological context, faunal assemblage and taphonomy of these fossils, as well as the Y/Ho ratio of their carbonates indicate an ancient freshwater or terrestrial ecology. This makes them the oldest freshwater/terrestrial brachyurans ever reported, extending the existence of freshwater crabs by 40 Ma. In this Campanian ecosystem, “true” crabs were associated to terrestrial vertebrates, including non-avian dinosaurs. Although they were likely well adapted to this environment, it cannot be decided whether *Dinocarcinus* was able to complete its life cycle in the freshwater habitat or/and had direct development. Its occurrence in the Late Cretaceous of Velaux-La Bastide Neuve, is an evidence for the independent colonizations of freshwater environments by multiple Brachyura clades over time, beside that of modern primary freshwater crabs (Potamoidea, Trichodactyloidea). It also supports the molecular clock estimation of an Early Cretaceous start for the radiation of Afrotropical freshwater crab taxa (just appearing in the Late Cretaceous), with the evidence of brachyuran crabs colonizing freshwater habitats as early as the Campanian.

## Material and Methods

The elementary composition of a brachyuran claw and of its surrounding matrix were investigated for their Y/Ho ratios. One gram of each was sampled on MMS.VBN.09.69e (claw/sediment). For the claw material, the basis of the propodus embedded in the matrix was mechanically sampled to preserve the connection between the claw and the turtle plate. Samples were microground and analysed for their composition in minor elements (in µg/g) normalized to PAAS, using ICMPS at the *Service d'Analyses des Roches et des Minéraux* of the CRPG, Vandoeuvre-lès-Nancy, France.

## Data Availability

All data needed to evaluate the conclusions in the paper are present in the paper and supplementary information.

## References

[1] Yeo, D. C. J. *et al*. In *Freshwater animal diversity assessment* 275–286 (Springer, 2008).

[2] Ortmann A (1896). Das System der Decapoden-Krebse. Zool. Jahrbücher, Abteilung für Syst. Geogr. und Biol. Thiere.

[3] Bott R (1970). Die Süßwasserkrabben von Europa, Asien und Australien und ihre Stammesgeschichte. Abh. senckenb. naturforsch. Ges.

[4] Milne Edwards H (1853). Mémoires sur la famille des Ocypodiens, suite. Ann. des Sci. Nat. Zool. Ser. 3.

[5] Schweitzer, C. E., Feldmann, R. M., Garassino, A., Karasawa, H. & Schweigert, G. Systematic list of fossil decapod crustacean species. in *Crustaceana Monographs* 230 pp, (Brill, 2010).

[6] Klaus S, Yeo DCJ, Ahyong ST (2011). Freshwater crab origins—laying Gondwana to rest. Zool. Anzeiger-A J. Comp. Zool..

[7] Klaus S, Magalhães C, Salas-Gismondi R, Gross M, Antoine P-O (2017). Palaeogene and Neogene brachyurans of the Amazon basin: a revised first appearance date for primary freshwater crabs (Brachyura, Trichodactylidae). Crustaceana.

[8] Szombathy K (1916). Die tertiären Formen der Gattung *Potamon* (*Telphusa*) und ihre paläarktischen Nachkommen. In Annales historico-naturales Musei Nationalis Hungarici, Budapest.

[9] Klaus S, Gross M (2010). Synopsis of the fossil freshwater crabs of Europe (Brachyura: Potamoidea: Potamidae). Neues Jahrb. für Geol. und Paläontologie-Abhandlungen.

[10] Hyžný, M. A freshwater crab *Potamon* (Brachyura: Potamidae) from the middle Miocene Lake Bugojno (Gračanica, Bosnia and Herzegovina), with notes on potamid taphonomy. *Palaeobiodiversity and Palaeoenvironments***in press**, 1–7

[11] Glaessner MF (1928). Die Dekapodenfauna des osterreichischen Jungtertiars. Jahrb. der Geol. Bundesanstalt.

[12] Glaessner, M. F. Dekapodenstudien. *Neues Jahrb. fur Mineral*. 137–176 (1929).

[13] Feldmann RM (2007). A new freshwater crab (Decapoda: Brachyura: Potamonautidae) from the Paleogene of Tanzania. Africa. Neues Jahrb. für Geol. und Paläontologie-Abhandlungen.

[14] Pasini, G. & Garassino, A. Unusual scaled preservation samples on freshwater decapods (Crustacea, Decapoda) from the Pleistocene (Late Cenozoic) of Turkey and Kazakistan. *Nat. Hist. Sci*. 13–18 (2011).

[15] Klaus, S. & Prieto, J. In Advances in freshwater decapod systematics and biology 161–172 (Brill, 2014).

[16] Ng, P. K. L. Fossil brachyuran crabs from the Jambusan Caves (Bau, Sarawak), collected by AH Everett in 1878–1879. *Scr. Geol* (2014).

[17] Klaus S, Böhme M, Schneider S, Prieto J, Phetsomphou B (2011). Evidence of the earliest freshwater decapod fossil from Southeast Asia (Crustacea: Decapoda: Brachyura). Raffles Bull. Zool..

[18] Klaus S (2017). A fossil freshwater crab from the Pliocene Tatrot Formation (Siwalik Group) in Northern India (Crustacea, Brachyura, Potamidae). Palaeoworld.

[19] Sternberg von R, Cumberlidge N, Rodriguez G (1999). On the marine sister groups of the freshwater crabs (Crustacea: Decapoda: Brachyura). J. Zool. Syst. Evol. Res..

[20] Tsang LM (2014). Evolutionary history of true crabs (Crustacea: Decapoda: brachyura) and the origin of freshwater crabs. Mol. Biol. Evol..

[21] Bott R (1972). Besiedlungsgeschichte und Systematik der Astaciden West-Europas unter besonderer Berücksichtigung der Schweiz. Rev. suisse Zool..

[22] Cumberlidge, N. & Ng, P. K. L. In *Decapod crustacean phylogenetics* 503–520 (CRC Press, 2016).

[23] Cincotta A (2015). Integrated paleoenvironmental reconstruction and taphonomy of a unique Upper Cretaceous vertebrate-bearing locality (Velaux, Southeastern France). PLoS One.

[24] Gradstein, F. M., Ogg, J., Schmitz, M. & Ogg, G. International Stratigraphic Chart. International Commission on Stratigraphy (2009).

[25] Garcia G, Vianey-Liaud M (2001). Dinosaur eggshells as biochronological markers in Upper Cretaceous continental deposits. Palaeogeogr. Palaeoclimatol. Palaeoecol..

[26] Garcia G, Amico S, Fournier F, Thouand E, Valentin X (2010). A new titanosaur genus (Dinosauria, Sauropoda) from the Late Cretaceous of southern France and its paleobiogeographic implications. Bull. la Société géologique Fr..

[27] Martin JE (2016). New specimens of *Allodaposuchus precedens* from France: intraspecific variability and the diversity of European Late Cretaceous eusuchians. Zool. J. Linn. Soc..

[28] Díez Díaz V (2018). The titanosaurian dinosaur *Atsinganosaurus velauciensis* (Sauropoda) from the Upper Cretaceous of southern France: new material, phylogenetic affinities, and palaeobiogeographical implications. Cretac. Res..

[29] Godefroit P (2017). Extreme tooth enlargement in a new Late Cretaceous rhabdodontid dinosaur from Southern France. Sci. Rep..

[30] Vullo R, Garcia G, Godefroit P, Cincotta A, Valentin X (2018). *Mistralazhdarcho maggii*, gen. et sp. nov., a new azhdarchid pterosaur from the Upper Cretaceous of southeastern France. J. Vertebr. Paleontol..

[31] Scotese, C. R. *Atlas of earth history*. (University of Texas at Arlington. Department of Geology. PALEOMAP Project, 2001).

[32] Csiki-Sava, Z., Buffetaut, E., Ősi, A., Pereda-Suberbiola, X. & Brusatte, S. L. Island life in the Cretaceous-faunal composition, biogeography, evolution, and extinction of land-living vertebrates on the Late Cretaceous European archipelago. *Zookeys* 1 (2015). 10.3897/zookeys.469.8439 PMC4296572 25610343

[33] Kato H, Karasawa H (1998). Pleistocene fossil decapod Crustacea from the Boso Peninsula, japan. Nat. Hist. Res. Spec. Issue.

[34] Portell, R. W. & Agnew, J. G. *Pliocene and Pleistocene decapod crustaceans*. (Florida Paleontological Society, 2004).

[35] Ando Y, Kawano S, Komatsu T, Niitani M (2016). Decapod crustaceans from the Pleistocene Oe Formation in Minamishimabara City, Nagasaki Prefecture, Japan. J. Foss. Res..

[36] Ando Y, Kawano S (2017). Decapods from the lower Pleistocene Masuda Formatio in Minamitane-cho, Kagoshima Prefecture, Japan. Bull. Mizunami Foss. Museum.

[37] Janssen AW, Müller P (1984). Miocene Decapoda and Mollusca from Ramsel (province of Antwerpen, Belgium), with a new crab genus and a new cephalopod species. Scr. Geol..

[38] Förster R (1979). Decapod crustaceans from the Middle Miocene (Badenian) deposits of southern Poland. Acta Geol. Pol..

[39] Förester R (1979). Decapod crustaceans from the Korytnica basin (Middle Miocene; Holy Cross Mountains, Central Poland). Acta Geol. Pol..

[40] Portell RW (2004). Eocene, Oligocene, and Miocene decapod crustaceans. Ann. Carnegie Museum.

[41] Schweitzer CE, Feldmann RM (2001). New Cretaceous and Tertiary decapod crustaceans from western North America. Bull. Mizunami Foss. Museum.

[42] Fraaije RHB, Van Bakel BWM, Iserbyt A, Jagt JWM (2011). New extinct Paguroidea (Crustacea, Decapoda, Anomura), with the first example of capsulated setae from the fossil record. Neues Jahrb. für Geol. und Paläontologie-Abhandlungen.

[43] Hyžný M, Fraaije RHB, Martin JE, Perrier V, Sarr R (2016). *Paracapsulapagurus poponguinensis*, a new hermit crab (Decapoda, Anomura, Paguroidea) from the Maastrichtian of Senegal. J. Paleontol..

[44] Devillez J, Charbonnier S (2019). Review of the Early and Middle Jurassic erymid lobsters (Crustacea: Decapoda). BSGF-Earth Sci. Bull..

[45] Devillez J, Charbonnier S, Veselská MK, Pezy J-P (2017). Review of the Late Cretaceous erymid lobsters (Crustacea: Decapoda) from the Western Tethys. Proc. Geol. Assoc..

[46] Beschin C, De Angeli A, Checchi A, Zarantonello G (2005). Crostacei eocenici di Grola presso Spagnago (Vicenza, Italia settentrionale). Stud. e Ric. Assoc. Amici del Museo, Mus. Civ. ‘G. Zannato’, Montecchio Magg..

[47] Hyžný M, Klompmaker AA (2015). Systematics, phylogeny, and taphonomy of ghost shrimps (Decapoda): a perspective from the fossil record. Arthropod Syst. phylogeny.

[48] Hyžný, M. & Kroh, A. Barremian decapod crustaceans from Serre de Bleyton (Drôme, SE France). Ann. des Naturhistorischen Museums Wien. Ser. A, Fur Mineral. und Petrogr. Geol. und Palaontologie, Anthropol. und Prahistorie **117**, 121 (2015). PMC4471114 26097276

[49] Jagt JWM, Bakel BWMVan, Artal P (2010). *Necrocarcinus ornatissimus* forir, 1887, and *Prehepatus werneri* Fraaye & Collins, 1987 (Upper Maastrichtian, the Netherlands) revisited, with notes on other Cretaceous dynomenid crabs (Decapoda, Brachyura). Crustac. Monogr..

[50] Spiridonov VA, Neretina TV, Schepetov D (2014). Morphological characterization and molecular phylogeny of Portunoidea Rafinesque, 1815 (Crustacea Brachyura): Implications for understanding evolution of swimming capacity and revision of the family-level classification. Zool. Anzeiger-A J. Comp. Zool..

[51] Manning RB, Holthuis LB (1981). West African brachyuran crabs. Sm. C. Zool..

[52] Ng PKL, Guinot D, Davie PJF, Systema Brachyurorum, Part I (2008). An annotated checklist of extant brachyuran crabs of the world. Raff. B. Zool..

[53] Lai JCY, Mendoza JCE, Guinot D, Clark PF, Ng PKL (2011). Xanthidae MacLeay, 1838 (Decapoda: Brachyura: Xanthoidea) systematics: A multi-gene approach with support from adult and zoeal morphology. Zool. Anz..

[54] Rathbun MJ (1923). Decapod crustaceans from the Upper Cretaceous of North Carolina. North Carolina Geol. Surv..

[55] Rathbun MJ (1917). New species of South Dakota Cretaceous crabs. Proc. United States Natl. Museum.

[56] Kesling RV, Reimann IG (1957). An Upper Cretaceous crab, *Avitelmessus grapsoideus* Rathbun. Contrib. from museum Paleontol. Univ. Michigan.

[57] Van Straelen V (1936). Crustacés Décapodes nouveaux ou peu connus de l’époque Crétacique. Bull. du Musée R. d’Histoire Nat. Belgique.

[58] Guinot D, Vega FJ, Van Bakel BWM (2008). Cenomanocarcinidae n. fam., a new Cretaceous podotreme family (Crustacea, Decapoda, Brachyura, Raninoidia), with comments on related families. Geodiversitas.

[59] Bosquet JAH (1854). Monographie des Crustaces fossiles du terrain Cretace du Duche de Limbourg. Verh. Uitg. door Comm. belast met het vervaardigen eener Geol. Beschrijv. en kaart van Ned..

[60] Jagt, J. W. M., Fraaije, R. H. B. & van Bakel, B. W. M. Decapod crustacean’odds and ends’ from the Maastrichtian type area (southeast Netherlands, northeast Belgium) *Distefania (*?) *vanrijsselti* n. sp. *Scr. Geol* (2014).

[61] Rathbun MJ (1935). Fossil Crustacea of the Atlantic and Gulf coastal plain. Geol. Soc. Am. Spec. Pap..

[62] Karasawa H (2008). Neogene and Quaternary ghost shrimps and crabs (Crustacea: Decapoda) from the Philippines. Bull. Natl. Museum Nat. Sci..

[63] Maury CJCretaceo (1930). da Parahyba do Norte. Serviço Geol. e Mineral. do Bras. Monogr..

[64] Ossó Àlex (2016). Eogeryon elegius n. gen. and n. sp. (Decapoda: Eubrachyura: Portunoidea), one of the oldest modern crabs from late Cenomanian of the Iberian Peninsula. Boletín de la Sociedad Geológica Mexicana.

[65] Vega FJ (2013). Morphology and size variation of a portunoid crab from the Maastrichtian of the Americas. J. South Am. Earth Sci..

[66] Ossó-Morales À, Artal P, Vega FJ (2010). New crabs (Crustacea, Decapoda) from the Upper Cretaceous (Campanian) of the Moyenne Moulouya, northeast Morocco. Rev. Mex. Ciencias Geológicas.

[67] Dietl GP, Vega FJ (2008). Specialized shell-breaking crab claws in Cretaceous seas. Biol. Lett..

[68] Colosi G (1923). Una specie fossile de Gerionide (Decapodi brachiuri). Bolettino della Soc. dei Nat. Napoli.

[69] Secretan S (1961). Une nouvelle espèce de xanthidés au Maroc: *Titanocarcinus meridionalis* nov. sp. *Notes Serv*. Géologique Maroc.

[70] Van Straelen V (1924). Description de Brachyoures montiens du Cominges. Bull. la Soc. Belgique Geol..

[71] Vega FJ (2001). Maastrichtian Crustacea (Brachyura: Decapoda) from the Ocozocuautla Formation in Chiapas, southeast México. J. Paleontol..

[72] Matheron, P. Notice sur les reptiles fossiles des dépôts fluvio-lacustres crétacés du bassin à lignite de Fuveau. (F. Savy, 1869).

[73] De Lapparent De Broin F, Murelaga X (1996). Une nouvelle faune de chéloniens dans le Crétacé supérieur européen. Comptes rendus de l'Académie des sciences. Série 2. Sciences de la terre et des planètes.

[74] Portis, A. Les chéloniens de la molasse vaudoise observé dans le Musée géologique de Lausanne. (Schuchardt, 1882).

[75] Nopcsa F (1928). Paleontological notes on Reptilia. 7. Classification of the Crocodilia. Geol. Hungarica, Ser. Palaeontol..

[76] Delfino M (2008). A complete skull of *Allodaposuchus precedens* Nopcsa, 1928 (Eusuchia) and a reassessment of the morphology of the taxon based on the Romanian remains. J. Vertebr. Paleontol..

[77] Puértolas-Pascual E, Canudo JI, Moreno-Azanza M (2014). The eusuchian crocodylomorph *Allodaposuchus subjuniperus* sp. nov., a new species from the latest Cretaceous (upper Maastrichtian) of Spain. Hist. Biol..

[78] Blanco A (2015). A new species of *Allodaposuchus* (Eusuchia, Crocodylia) from the Maastrichtian (Late Cretaceous) of Spain: phylogenetic and paleobiological implications. PeerJ.

[79] Blanco A, Puértolas-Pascual E, Marmi J, Vila B, Sellés AG (2014). *Allodaposuchus palustris* sp. nov. from the Upper Cretaceous of Fumanya (South-Eastern Pyrenees, Iberian Peninsula): systematics, palaeoecology and palaeobiogeography of the enigmatic allodaposuchian crocodylians. PLoS One.

[80] Taplin LE, Grigg GC, Harlow P, Ellis TM, Dunson WA (1982). Lingual salt glands in *Crocodylus acutus* and *C. johnstoni* and their absence from *Alligator mississipiensis* and *Caiman crocodilus*. J. Comp. Physiol. B Biochem. Syst. Environ. Physiol..

[81] Pérez-García A (2017). The Iberian fossil record of turtles: an update. J. Iber. Geol..

[82] Tong, H., Gaffney, E. S. & Buffetaut, E. *Foxemys*, a new side-necked turtle (Bothremydidae, Pelomedusoides) from the late Cretaceous of France. American Museum novitates; no. 3251. (1998).

[83] Gaffney ES, Tong H, Meylan PA (2006). Evolution of the side-necked turtles: the families Bothremydidae, Euraxemydidae, and Araripemydidae. Bull. Am. Museum Nat. Hist..

[84] Pérez-García A, Ortega F (2018). Identification of the French Upper Cretaceous bothremydid turtle *Foxemys mechinorum* in the Spanish record. Geobios.

[85] Joyce WG, Chapman SD, Moody RTJ, Walker CA (2011). The skull of the solemydid turtle *Helochelydra nopcsai* from the Early Cretaceous of the Isle of Wight (UK) and a review of Solemydidae. Spec. Pap. Palaeontol..

[86] Case G, Cappetta H (2004). Additions to the elasmobranch fauna from the Upper Cretaceous of New Jersey (Middle Maastrichtian, Navesink Formation). Palaeovertebrata.

[87] Cuny, G., Suteethorn, V. & Buffetaut, E. Freshwater hybodont sharks from the Lower Cretaceous of Thailand. *Biol. Conserv. Freshw. Elasmobranchs, Am. Fish. Soc. Bethesda, Maryl*. 15–26 (2004).

[88] Cavin L, Valentin X, Garcia G (2016). A new mawsoniid coelacanth (Actinistia) from the Upper Cretaceous of Southern France. Cretac. Res..

[89] Webb GE, Kamber BS (2000). Rare earth elements in Holocene reefal microbialites: a new shallow seawater proxy. Geochim. Cosmochim. Acta.

[90] Nothdurft LD, Webb GE, Kamber BS (2004). Rare earth element geochemistry of Late Devonian reefal carbonates, Canning Basin, Western Australia: confirmation of a seawater REE proxy in ancient limestones. Geochim. Cosmochim. Acta.

[91] Mavotchy NO (2016). The role of the early diagenetic dolomitic concretions in the preservation of the 2.1-Ga paleoenvironmental signal: The Paleoproterozoic of the Franceville Basin, Gabon. Comptes Rendus Géoscience.

[92] Høgdhal, O., Melsom, S. & Bowen, V. In *Trace Inorganics In**Water* 308–325 (ACS Publications, 1968).

[93] Bolhar R, Kamber BS, Moorbath S, Fedo CM, Whitehouse MJ (2004). Characterisation of early Archaean chemical sediments by trace element signatures. Earth Planet. Sci. Lett..

[94] Nozaki Y, Lerche D, Alibo DS, Tsutsumi M (2000). Dissolved indium and rare earth elements in three Japanese rivers and Tokyo Bay: evidence for anthropogenic Gd and. Geochim. Cosmochim. Acta.

[95] Locatelli, E. R. Experimental taphonomy of decapod crustaceans: assessing the effects of pre-burial processes. In *Geological Society of America Annual Meeting. Abstracts with programm* 102–137 (2012).

[96] Klompmaker AA, Portell RW, Frick MG (2017). Comparative experimental taphonomy of eight marine arthropods indicates distinct differences in preservation potential. Palaeontology.

[97] Gallagher WB, Parris DC, Spamer EE (1986). Paleontology, biostratigraphy, and depositional environments of the Cretaceous-Tertiary transition in the New Jersey Coastal Plain. The Mosasaur.

[98] Gallagher WB (1993). The Cretaceous/Tertiary mass extinction event in the northern Atlantic Coastal Plain. The Mosasaur.

[99] Kuehne D (1993). Further examination of the Woodbury and Englishtown Formations in Camden County and adjacent areas, New Jersey. The Mosasaur.

[100] Schweitzer CE (2003). Mangrove-dwelling crabs (Decapoda: Brachyura: Necrocarcinidae) associated with dinosaurs from the Upper Cretaceous (Cenomanian) of Egypt. J. Paleontol..

[101] Bott R (1955). Die Süßwasserkrabben von Afrika (Crust., Decap.) und ihre Stammesgeschichte. Ann. du Mus. R. du Congo Belge - Ser. C.

[102] Schubart CD, Ng PKL (2008). A new molluscivore crab from Lake Poso confirms multiple colonization of ancient lakes in Sulawesi by freshwater crabs (Decapoda: Brachyura). Zool. J. Linn. Soc..

[103] Chin K, Feldmann RM, Tashman JN (2017). Consumption of crustaceans by megaherbivorous dinosaurs: Dietary flexibility and dinosaur life history strategies. Sci. Rep..

[104] Kirkland, J. I. An inventory of paleontological resources in the lower Wahweap Formation (lower Campanian), Southern Kaiparowits plateau, Grand staircase ľ Escalante National monument, Utah. In *2005 Salt Lake City Annual Meeting* (2005).

[105] DeBlieux, D. D. *et al*. Paleontological overview and taphonomy of the middle Campanian Wahweap Formation in Grand Staircase-Escalante National Monument. *Top Gd. Staircase Late Cretac. South. Utah* 563–587 (2013).

[106] de Broin, F. *et al*. Nouvelles découvertes de vertébrés maestrichtiens dans le gisement de Fox-Amphoux (Var). *8e Réunion Annu. des Sci. la Terre, Marseille* 68 (1980).

[107] Codrea V, Solomon A, Fărcaș C, Barbu O (2013). On some local restricted Maastrichtian environments of the “Hațeg Island” (Transylvania, Romania). Bull. Geol. Soc. Greece.

[108] De Grave S, Cai Y, Anker A (2008). Global diversity of shrimps (Crustacea: Decapoda: Caridea) in freshwater. Hydrobiologia.

[109] Kemp S (1925). Notes on Crustacea Decapoda in the Indian Museum XVII. On various Caridae. *Rec. Indian*. Museum.

[110] Abele LG (1970). Semi-terrestrial shrimp (*Merguia rhizophorae*). Nature.

[111] Crandall, K. A. & Buhay, J. E. In *Freshwater animal diversity assessment* 295–301 (Springer, 2007).

[112] Holmes SJ (1904). On some new or imperfectly known species of west American Crustacea. Proc. Calif. Acad. Sci. (3, Zool..

[113] Dworschak, P. C. First record of *Lepidophthalmus tridentatus* (von Martens, 1868)(Callianassidae) from the Philippines. *Ann. des Naturhistorischen Museums Wien. Ser. B für Bot. und Zool*. 121–130 (2006).

[114] White, A. Descriptions of two species of Crustacea belonging to the families Callianassidae and Squillidae. In *Proceedings of the Scientific Meetings of the Zoological Society of London* 42–44 (1861).

[115] Vanhöffen E (1911). Über die Krabben, denen Kamerun seinen Namen verdankt. Sitzungsberichte der Gesellschaft Naturforschender Freunde zu Berlin.

[116] Dana JD (1852). Conspectus crustaceorum, quae in orbis terrarum circumnavigatione, Carolo Wilkes, e classe Reipublicae foederatae duce, lexit et descripsit Jacobus D. Dana. Pars II. Proc. Am. Acad. Arts Sci. 2nd Ser..

[117] Bond-Buckup G, Buckup L (1994). A família Aeglidae (Crustacea, Decapoda, Anomura). Arq. Zool..

[118] Perez-Losada M, Jara CG, Bond-Buckup G, Porter ML, Crandall KA (2002). Phylogenetic position of the freshwater anomuran family Aeglidae. J. Crustac. Biol..

[119] Pérez-Losada M, Bond-Buckup G, Jara CG, Crandall KA (2004). Molecular systematics and biogeography of the southern South American freshwater “crabs” Aegla (Decapoda: Anomura: Aeglidae) using multiple heuristic tree search approaches. Syst. Biol..

[120] Cumberlidge N (2009). Freshwater crabs and the biodiversity crisis: importance, threats, status, and conservation challenges. Biol. Conserv..

[121] Haan de, W. In Fauna japonica, sive, Descriptio animalium, quae in itinere per Japoniam, jussu et auspiciis, superiorum, qui summum in India Batava imperium tenent, suscepto, annis 1823-1830. Volume 1: Crustacea. (ed. Siebold, P. F. V.; Haan, W. D.; Schlegel, H. & Temminck, C. J.) 243 (1833).

[122] Milne Edwards, H. In *Dictionnaire Classique d’Histoire Naturelle* (ed. Bory de Saint-Vincent, J.-B. G. M.) 16 (Ray et Gravier; Amable Gobin et Cie, 1830).

[123] Ng, P. K. L. The freshwater crabs of Peninsular Malaysia and Singapore (1988).

[124] Ng, P. K. L. In *Freshwater invertebrates of the* Malaysian *region* (eds. Yong, H. S. & Yule, C. M.) 311–336 (Akademi Sains Malaysia, 2004).

[125] Davie PJF, Ng PKL (2007). A new genus for cave-dwelling crabs previously assigned to *Sesarmoides* (Crustacea: Decapoda: Brachyura: Sesarmidae). Raffles Bull. Zool. Suppl..

[126] De Man, J. G. In Zoologische Ergebnisse einer Reise in Niederlandisch OstIndien (ed. Weber, M.) 165–527 (1892).

[127] Feldmann, R. M., Grande, L., Birkhimer, C. P., Hannibal, J. T. & McCoy, D. L. Decapod fauna of the Green River formation (Eocene) of Wyoming. *J. Paleontol*. 788–799 (1981).

[128] Hasiotis ST, Mitchell CE (1993). A comparison of crayfish burrow morphologies: Triassic and Holocene fossil, paleo‐and neo‐ichnological evidence, and the identification of their burrowing signatures. Ichnos An Int. J. Plant Anim..

[129] Rabadà i Vives, D. Crustáceos decápodos lacustres de las calizas litográficas del Cretácico inferior de España: Las Hoyas (Cuenca) y el Montsec de Rúbies (Lleida) (1993).

[130] Taylor RS, Schram FR, Shen Y-B (1999). A new crayfish family (Decapoda: Astacida) from the Upper Jurassic of China, with a reinterpretation of other Chinese crayfish taxa. Paleontol. Res..

[131] Martin AJ (2008). Fossil evidence in Australia for oldest known freshwater crayfish of Gondwana. Gondwana Res..

[132] Beurlen K (1950). Alguns restos de crustáceos decápodes d’água doce fósseis no Brasil. An. da Acad. Bras. ciencias.

[133] Houša V (1956). Bechleja inopinata ng, n. sp. ein neuer Krebs aus dem bohmischen Tertiar (Decapoda, Palaemonidae). Ustred Ust. Geol..

[134] Martins-Neto RG, Mezzalira S (1991). Revisão dos Palemonídeos Terciários Brasileiros (Crustacea, Caridea) com descrição de novos taxa. An. Acad. Bras. Cienc..

[135] Garassino A, Yanbin S, Schram FR, Taylor RS (2002). *Yongjicaris zhejiangensis* n. gen. n. sp.(Crustacea, Decapoda, Caridea) from the Lower Cretaceous of Zhejiang Province, China. *Bull. Mizunami Foss*. Museum.

[136] Feldmann RM (1984). *Haumuriaegla glaessneri* n. gen. and sp.(Decapoda; Anomura; Aeglidae) from Haumurian (late Cretaceous) rocks near Cheviot, New Zealand. New Zeal. J. Geol. Geophys..

[137] De Angeli, A. & Caporiondo, F. *Alontecarcinus buratoi* n. gen., n. sp. (Decapoda, Brachyura, Potamonidae) un nuovo crostaceo dacqua dolce dellEocene (Bartoniano) di Alonte (Monti Berici, Vicenza, Italia settentrionale). *Bolletino del Mus. di Stor. Nat. di Verona* in press.

[138] Ma KY (2019). Phylogenomic analyses of brachyuran crabs support early divergence of primary freshwater crabs. Mol. Phylogenet. Evol..

[139] Garassino A, Pasini G, Dutheil DB (2006). *Cretapenaeus berberus* n. gen., n. sp. (Crustacea, Decapoda, Penaeidae) from the Late Cretaceous (Cenomanian) of southeastern Morocco. Atti della Soc. Ital. di Sci. Nat. e del Mus. Civ. di Stor. Nat. di Milano.

[140] Breinholt, J., Pérez-Losada, M. & Crandall, K. A. The timing of the diversification of the freshwater crayfishes. *Decapod Crustac. phylogenetics* 343–356 (2009).

[141] Peach, B. N. Monograph on the higher Crustacea of the Carboniferous rocks of Scotland. *Mem. Geol. Surv. Gt. Britain, Palaeontol*. 1–82 (1908).

[142] Gueriau P, Charbonnier S, Clément G (2014). First decapod crustaceans in a Late Devonian continental ecosystem. Palaeontology.

[143] Jones WT, Feldmann RM, Schram FR, Schweitzer CE, Maguire EP (2016). The proof is in the pouch: *Tealliocaris* is a peracarid. Palaeodiversity.

[144] Bate CS (1868). On a new genus, with four new species of freshwater prawns. Proc. Zool. Soc. London.

[145] Botello A, Alvarez F (2013). Phylogenetic relationships among the freshwater genera of palaemonid shrimps (Crustacea: Decapoda) from Mexico: evidence of multiple invasions?. Lat. Am. J. Aquat. Res..

[146] Crandal KA, Harris DJ, Fetzner JW (2000). The monophyletic origin of freshwater crayfish estimated from nuclear and mitochondrial DNA sequences. Proc. R. Soc. London. Ser. B Biol. Sci..

[147] Klaus S, Schubart CD, Brandis D (2006). Phylogeny, biogeography and a new taxonomy for the Gecarcinucoidea Rathbun, 1904 (Decapoda: Brachyura). Org. Divers. Evol..

[148] Sternberg von, R. & Cumberlidge, N. In *Advances in Decapod Crustacean Research* 21–39 (Springer, 2001).

[149] Daniels SR, Cumberlidge N, Pérez-Losada M, Marijnissen SAE, Crandall KA (2006). Evolution of Afrotropical freshwater crab lineages obscured by morphological convergence. Mol. Phylogenet. Evol..

[150] Le Loeuff J (2005). Romanian Late Cretaceous dinosaurs: big dwarfs or small giants?. Hist. Biol..

